# Tau Protein and β-Amyloid Associated with Neurodegeneration in Myelin Oligodendrocyte Glycoprotein-Induced Experimental Autoimmune Encephalomyelitis (EAE), a Mouse Model of Multiple Sclerosis

**DOI:** 10.3390/biomedicines12122770

**Published:** 2024-12-05

**Authors:** Grażyna Pyka-Fościak, Ewa Jasek-Gajda, Bożena Wójcik, Grzegorz J. Lis, Jan A. Litwin

**Affiliations:** Department of Histology, Jagiellonian University Medical College, Kopernika 7, 31-034 Krakow, Poland; ewa.jasek@uj.edu.pl (E.J.-G.);

**Keywords:** tau protein, p-tau protein, β-amyloid, inflammation, neurodegeneration, EAE, multiple sclerosis

## Abstract

Background: The levels of β-amyloid precursor protein (β-APP), tau protein, and phosphorylation of tau (p-tau) protein were examined by quantitative immunohistochemistry in the spinal cord sections of mice suffering from experimental autoimmune encephalomyelitis (EAE) in the successive phases of the disease: onset, peak, and chronic. Methods: EAE was induced in C57BL/6 mice by immunization with MOG35–55 peptide. The degree of pathological changes was assessed in cross-sections of the entire spinal cord. Results: β-APP expression was observed in the white matter and colocalized with some Iba-1-positive macrophages/microglia. It increased in the peak phase of EAE and remained at the same level in the chronic phase. During the onset and peak phases of EAE, expression of tau protein was observed in nerve fibers and nerve cell perikaryons, with a predominance of nerve fibers, whereas in the chronic phase, tau was labeled mainly in the perikaryons of nerve cells, with its content significantly decreased. P-tau immunostaining was seen only in nerve fibers. Conclusions: The expression of p-tau increased with the progression of EAE, reaching the maximum in the chronic phase. The correlation between these proteins and neurodegeneration/neuroinflammation highlights their potential roles in the progression of neurodegenerative mechanisms in MS.

## 1. Introduction

The most common central nervous system (CNS) inflammatory disease is multiple sclerosis (MS), modeled in mice by experimental autoimmune encephalomyelitis (EAE). EAE shows a very close similarity in pathological changes to human MS and is widely recognized as an animal model in MS research [1]. At the pathological level, MS/EAE is characterized by the infiltration of inflammatory lymphocytes directed against myelin antigens into the CNS (brain, spinal cord, and optic nerve), triggering a chronic inflammatory response, which results in the stripping of the myelin sheath from myelinated axons (demyelination) and, ultimately, axonal degeneration [2,3].

Post-translational changes in neuronal proteins may cause destabilization of the axonal cytoskeleton, negatively affecting the physiology of neurons. Proteins associated with neurodegenerative diseases, such as β-amyloid (β-APP) and tau, can form insoluble aggregates that play a role in neuronal pathology and degeneration. Tracking them as biomarkers of neurodegeneration over time is increasingly being considered [4]. “The amyloid cascade hypothesis” postulates that the formation of β-amyloid deposits in neural tissue is the initiating event in Alzheimer disease (AD) pathogenesis, resulting in the subsequent formation of tau tangles, neuronal loss, and dysfunction, as well as cognitive decline [5,6].

An increase in β-APP levels is hypothesized to be the key event in AD and in other neurodegenerative disorders, including MS, that triggers tau pathology, followed by neuronal death and, ultimately, disease [7,8]. Moreover, it is also postulated that once tau pathology occurs, anti-β-APP therapies are no longer effective because the disease will progress independently of the treatment [9].

Amyloidogenic proteins detected in MS lesions and EAE models are associated with neuronal damage [10,11]. The histopathological hallmark in neurodegenerative diseases, including the EAE model, is an abnormal form of tau aggregates, which stick together into thread-like structures called neurofibrillary tangles. Tau is a type of microtubule-associated protein that is mainly concentrated in axons and is responsible for microtubule assembly and stability, directly interacting with proteins involved in microtubule-dependent transport. Abnormal phosphorylation of tau (p-tau) leads to decreased microtubule stability and can result in the formation of potentially neurotoxic aggregates. Ineffective removal of tangles and their accumulation damages axonal transport, disrupting the function of neurons [7,9]. The intracellular accumulation of tau aggregates underlies disorders known as “tauopathies”.

The other histopathological symptom of neurodegeneration is the extracellular deposition of β-APP amyloid plaques formed as a result of APP proteolysis between neurons, contributing to inflammatory changes and the production of pro-inflammatory cytokines [12]. Moreover, in MS lesions, the observed accumulation of amyloid correlates with different stages of the disease [12,13,14].

Amyloid-forming molecules, β-APP and abnormal tau, are generally regarded as harmful and as the main pathogenic culprits. However, in neurodegenerative diseases, including MS/EAE, some forms of APP and tau proteins may have an immunosuppressive effect, reducing the level of inflammatory cytokines and the degree of paralysis, thus showing unexpected benefits in neuroinflammatory conditions [9,12].

In this study, we investigated two major pathological hallmarks in the EAE model: extracellular deposition of β-APP plaques between neurons and intracellular aggregates of neurofibrillary tangles of abnormal tau proteins inside the neurons. We examined the immunohistochemical expressions of tau/p-tau and β-APP in the successive phases of EAE and compared them with quantitatively assessed standard histopathological parameters of MS/EAE, such as inflammatory infiltration, demyelination, and axonal damage in spinal cord samples. The involvement of tau/p-tau and beta-APP in the EAE model could provide new insights into the mechanisms driving neurodegeneration in MS, offering potential therapeutic interventions targeting these proteins.

## 2. Materials and Methods

### 2.1. Experimental Animals

All studied animals were handled in compliance with Council Directive 2010/63/EU of the European Parliament and of the Council of 22 September 2010 on the protection of animals used for scientific purposes. All mice experiments were approved by the Local Ethics Committee of the Jagiellonian University Medical College, Krakow, Poland (Permissions 118/2015 and 274/2018).

Female C57BL/6 mice (*n* = 30), weight 19–24 g, aged 10–11 weeks, purchased from the Center of Experimental Medicine, Medical University of Bialystok (strain imported from Jackson Laboratory, Bialystok, Poland), were used in the study. Before the experiment, animals were habituated for at least 1 week and then were immunized. Mice were housed in an animal house at the Jagiellonian Centre for Experimental Therapeutics (JCET), Krakow under a 12 h light–dark cycle in a temperature-controlled environment (22 ± 2 °C, 55 ± 10% humidity). Standard irradiated laboratory chow and water were available ad libitum. 

### 2.2. EAE-Inducted Mice

EAE was induced using a protocol and materials provided by Hooke Laboratories (Lawrence, MA, USA). On day 0, mice (*n* = 15) were injected subcutaneously with 200 μL of Hooke Kits™ EAE Emulsion (Hooke Laboratories, Lawrence, MA, USA) containing MOG_35–55_ peptide emulsified in complete Freud’s adjuvant (CFA) containing 2 mg/mL Mycobacterium tuberculosis (100 µL behind the neck and 100 µL in hind flank). In addition, on days 0 and 1, mice also received an intraperitoneal injection of 340 µL of *Bordatella pertussis* toxin (PTx) dissolved in phosphate-buffered saline (PBS) 2 h after the administration of the emulsion and again 24 h later. Control mice (*n* = 15) were injected with CFA and PTx only according to the same schedule.

### 2.3. Evaluation of EAE and Phases of EAE Disease

The clinical symptoms of EAE were scored following protocols provided by Hooke Laboratories and as reported previously [15]. All mice were weighed and examined daily in search of clinical signs of EAE. The symptoms of EAE were monitored, and clinical scores were recorded daily for 30 days after disease induction using a scale ranging from 0 to 3: (0) no clinical symptoms; 0.5—limp tip of tail; 1—flaccid tail; 1.5—flaccid tail and hind leg inhibition; 2—flaccid tail and weakness of hind legs; 2.5—flaccid tail and dragging of hind legs; and 3—flaccid tail and complete paralysis of hind legs. Three distinct phases of EAE were distinguished, i.e., onset (day 14), peak (day 19), and chronic (day 30). The immunized mice (*n* = 15) were sacrificed on these days (*n* = 5 per phase). Non-immunized, control mice (*n* = 15) were sacrificed on days corresponding to the previously mentioned phases (*n* = 5 per phase). The cumulative clinical score was calculated as a sum of daily clinical scores. 

### 2.4. Sample Collection

To collect spinal cords, mice were anesthetized with a ketamine/xylazine cocktail (100 mg/kg and 10 mg/kg, respectively, administered intraperitoneally). Animals were perfused transcardially with ice-cold PBS for 5 min and then with 4% paraformaldehyde for 10 min. Next, spinal cords were removed, fixed in 4% paraformaldehyde at 4 °C for 4 h, and stored overnight in 5% sucrose solution in PBS at 4 °C. Next, samples were embedded in an optimal cutting temperature mixture (OCT compound, Shandon Cryomatrix, Thermo Fisher Scientific, Rockford, IL, USA) and snap-frozen at −80 °C. Serial 10 µm thick cryosections were cut at 50 μm intervals, collected on poly-L-lysine-coated slides, and air-dried. The sections were fixed with acetone for 20 min and air-dried again. Histological analysis was performed on sections of the lumbar spinal cords, an area frequently and rapidly affected by EAE.

### 2.5. Histopathological Staining

To analyze histopathological changes, hematoxylin–eosin (H&E), Luxol Fast Blue (LFB), Bielschowsky silver impregnation (BSI), and Congo Red (CR) stainings were performed. H&E staining reveals inflammatory cell infiltration, LFB shows demyelination, and BSI detects axonal damage [15]. CR is the standard stain used to identify amyloid material and neurofibrillary tangles in nerve cells [16].

### 2.6. Immunohistochemical Staining

The spinal cord tissue sections were preincubated for 30 min in PBS containing 5% normal goat serum (Sigma-Aldrich, St. Louis, MO, USA), 0.1% bovine serum albumin, 0.05% thimerosal, 0.01% sodium azide, 0.5% Triton X-100, and 2% dry milk. Subsequently the sections were incubated overnight with primary antibodies: rabbit anti-β-APP for β-amyloid peptide (Thermo Fisher Scientific, Rockford, IL, USA); 1:100 cat. # 36-6900), rabbit anti-tau for tau protein (1:300; Thermo Fisher Sci., Rockford, IL, USA cat. #PA5-29610), rabbit anti-phospho-tau (Ser396) for p-tau protein (1:500; Thermo Fisher Sci., Rockford, IL, USA cat. #44-752G), rat anti-CD45 for total leukocytes (1:100; Thermo Fisher Sci., Rockford, IL, USA cat. #MA1-81247), and rabbit anti-Iba1 for activated macrophages and microglial cells (1:100; Thermo Fisher Sci., Rockford, IL, USA cat. #MA5-50414). After a rinse in PBS, sections were incubated for 90 min with the secondary Cy3-conjugated goat anti-rat antiserum (Jackson IR, West Grove, PA, USA; 1:300, cat. #112-165-167), Cy3-conjugated goat anti-rabbit antibodies (Jackson IR, West Grove, PA, USA; 1:300; cat. # 111-165-144), or goat anti-rabbit Alexa488-conjugated antibodies (Jackson IR, West Grove, PA, USA; 1:100, cat. #111-545-144). Finally, the sections were washed with PBS and mounted in a glycerol/PBS solution. DAPI staining (Thermo Fisher Sci., Rockford, IL, USA; 1.5 µg/mL; cat. # 62248) was used to visualize cell nuclei.

### 2.7. Morphometry

The degree of pathological changes was assessed in cross-sections of the entire spinal cord, including gray and white matter. The degree of inflammation was evaluated in two ways: as the number of inflammatory lesions and as the percentage of infiltrated areas relative to the total area of the entire spinal cord (darker-colored cellular infiltrates). Demyelination and axonal loss were observed as lighter areas in the white matter, and the percentage of such areas reflected its degree. In immunostained sections, the percentage of the fluorescent area relative to the total cross-sectional area of the spinal cord, was calculated for each antigen. A total of 50 sections were counted per experimental group/phase (*n* = 5).

### 2.8. Image Collection and Statistical Analysis

Sections were examined with an Olympus BX50 bright-field/epifluorescence microscope (Olympus, Tokyo, Japan). Images were captured with an Olympus DP71 digital CCD camera (1360 × 1024 pixels), stored as TIFF files, and processed for quantitative analysis using ImageJ software (Version 1.51, NIH, Bestheda, MD, USA). 

The data were presented as means ± standard error of the mean (SEM). The statistical significance of quantitative data was assessed using one-way analysis of variance (ANOVA), followed by Bonferroni’s multiple comparisons post hoc test; *p* < 0.05 was defined as statistically significant. The Pearson correlation coefficient test was used to analyze the relationship between histological parameters with β-APP/tau/p-tau expression for EAE mice. All analyses were performed using Prism 5.0 software (GraphPad, La Jolla, CA, USA).

## 3. Results

### 3.1. Characterization of EAE Severity

**Figure 1:**
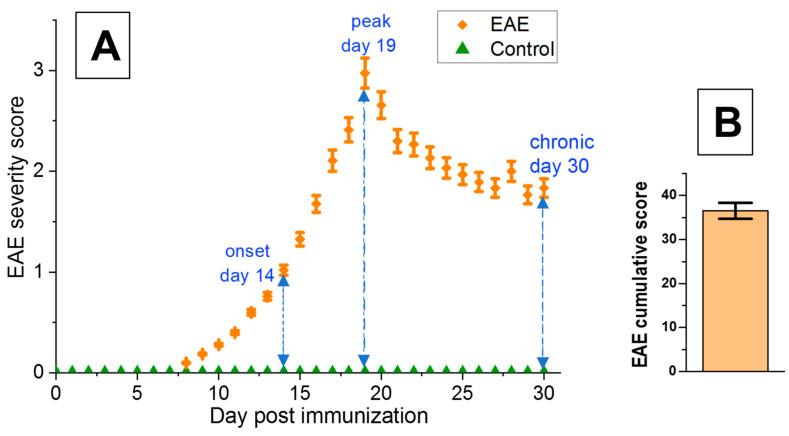
The progression of clinical severity in the successive phases of EAE: on day 14—onset phase, day 19—peak phase, and day 30—chronic phase (the dashed lines). No clinical scores for control mice. Each data point represents the average score (+/−S.E.M.) for disease severity at the indicated post-immunization time points (**A**). EAE cumulative score as the sum of individual EAE courses (+/−S.E.M.) (**B**).

All immunized mice developed neurological symptoms of EAE. On the basis of the applied scoring system, the clinical results of EAE were analyzed in three different phases of EAE disease: onset, peak, and chronic. As shown in Figure 1A, no signs of EAE were observed between 0–7 days post-immunization (dpi). The first symptoms were observed on dpi 8. The onset phase of the disease was registered on dpi 14 (the mean score was 1.02 ± 0.05), the peak phase on dpi 19 (the mean score 2.97 ± 0.15), and recovery or chronic phase on dpi 30 (the mean score 1.83 ± 0.09) (Figure 1A, Table 1). The cumulative score was 36.52 ± 1.83 (Figure 1B, Table 1). Control, non-immunized mice did not show any symptoms of the disease (Figure 1A).

### 3.2. Histopathological Changes in the Spinal Cord

**Figure 2:**
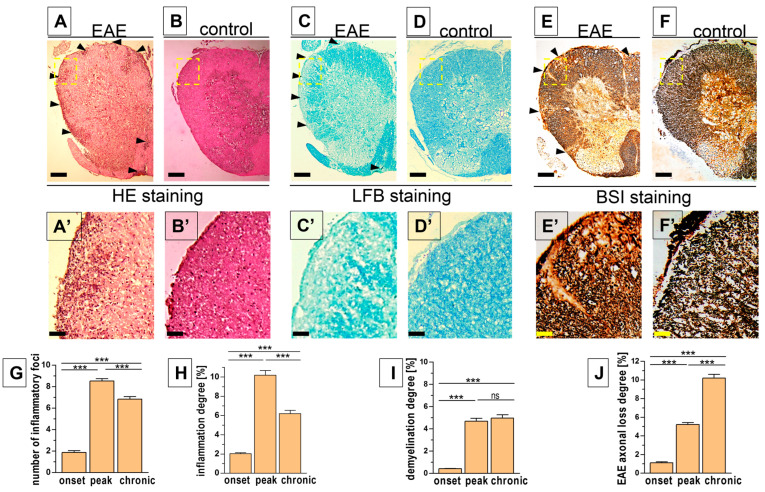
Quantitative histology of spinal cord samples of EAE and control mice. Hematoxylin and eosin (H&E) staining reveals inflammatory cell infiltration (**A**,**B**,**A’**,**B’**), Luxol Fast Blue (LFB) shows demyelination (**C**,**D**,**C’**,**D’**), and Bielschovsky silver impregnation (BSI) visualizes axonal loss (**E**,**F**,**E’**,**F’**) in the spinal cord on day 19 after immunization for EAE (**A**,**C**,**E**) and in control mice, which did not show any pathologies (**B**,**D**,**F**,**B’**,**D’**,**F’**). Diagrams show the number of inflammatory plaques (**G**), degree of inflammation (**H**), demyelination (**I**), and axonal loss (**J**) in three phases of EAE progression. Areas enclosed within yellow dashed squares (**A**–**F**) are magnified bellow, respectively (**A’**–**F’**). To assess the degree of pathological changes, the areas occupied by inflammatory infiltrate (**A**,**A’**), pale areas of demyelination (**C**,**C’**), and the brighter area for axonal loss (**E**,**E’**) (all marked by arrowheads) were quantified as the percentage of the cross-sectioned spinal cord section area (**G**–**J**). The data are expressed as means ± SEM; *n* = 5 per group. Statistical significance was verified using ANOVA with Bonferroni’s multiple comparisons test at a 0.05 confidence level (*** *p* < 0.001). Scale bars: (**A**–**F**) = 200 µm, insets (**A’**–**F’**) = 50 µm.

**Figure 3:**
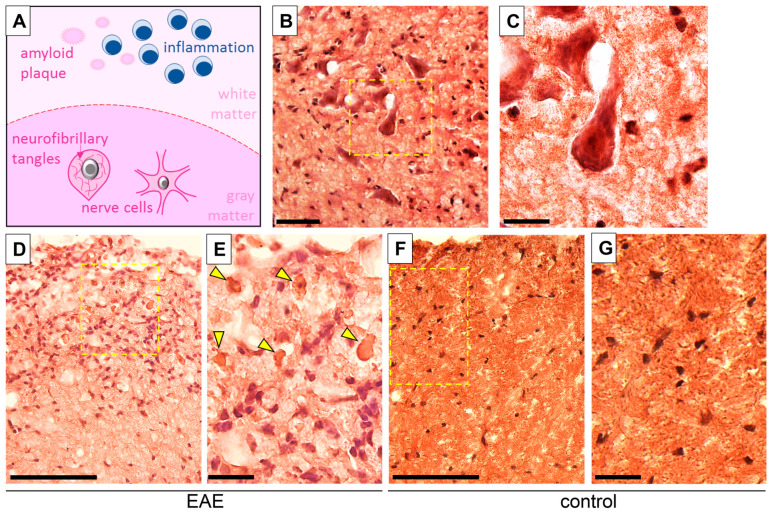
A schematic illustration depicting pathological changes in the white and gray matter of the spinal cord: inflammation, amyloid aggregation, and intracellular neurofibrillary tangles in nerve cells (**A**). Congo red staining visualizes intraneuronally accumulated tau ((**B**), inset (**C**)) and amyloid deposits (arrowheads) associated with inflammatory conditions (high density of leukocyte nuclei) in the chronic phase of EAE ((**D**), inset (**E**)) and the spinal cord for control ((**F**), inset (**G**)). Areas enclosed within yellow dashed squares (**B**,**D**,**F**) are magnified on the right, respectively (**C**,**E**,**G**). Scale bars: (**B**,**D**,**F**) = 100 µm, and insets (**C**,**E**,**G**) = 20 µm.

Histological stainings used to demonstrate pathological changes in the spinal cord were followed by a quantitative analysis of the degree of inflammation (H&E staining), demyelination (LFB staining), and axonal damage (BSI staining) (Figure 2A–F,A’–F’), as well as extracellular amyloid aggregation and intracellular neurofibrillary tangles in nerve cells (CR staining, Figure 3).

In EAE mice, the temporal pattern of inflammatory parameters in the spinal cord was similar to that of the severity of EAE expressed by clinical indices. Both the number and area of inflammatory foci showed a marked increase in the onset phase, reached a maximum in the peak phase, and decreased to the lowest values in the chronic phase (Figure 2G,H, Table 1). In case of demyelinating lesions, a similar increase was observed between the onset and peak phases. However, in the chronic phase of EAE progression, the demyelination remained at a similar level (Figure 2I, Table 1). A gradual increase in axonal loss was observed from the onset to the chronic phase during EAE progression (Figure 2J, Table 1). No histological signs of inflammatory infiltration, demyelination, and axonal loss were observed in control mice (Figure 2B,D,F,B’,D’,F’). 

CR staining was performed to confirm the presence of large extracellular amyloid plaques (length 5–10 µm) in the spinal cord tissue. Amyloid deposits between inflammatory cells in the white matter of the spinal cord were observed in the peak and chronic phases of EAE (Figure 3D,E). Intracellular neurofibrillary tangles accumulated in cell bodies of neurons were also visible after CR staining (Figure 3B,C). No such histological changes were observed in the control groups (Figure 3F,G).

### 3.3. Intercellular β-APP Aggregation and Expression Level

**Figure 4:**
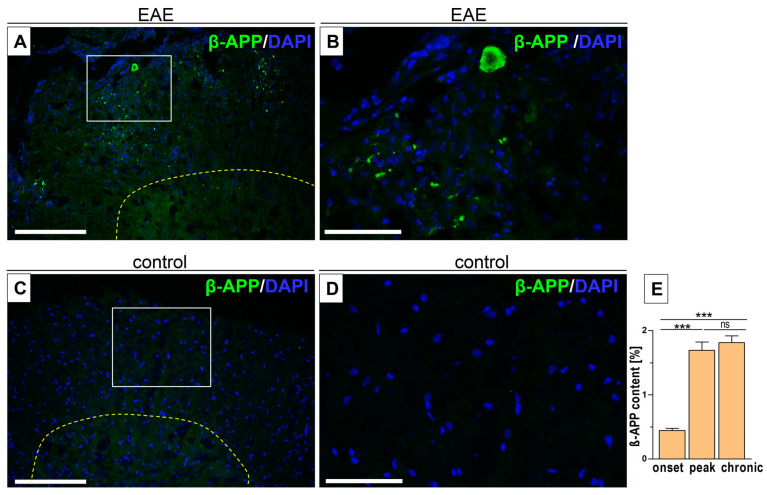
Intercellular β-amyloid (β-APP) aggregation associated with inflammatory infiltration (DAPI staining of the nuclei) ((**A**), inset (**B**)). No β-APP deposition in control mice ((**C**), inset (**D**)) Quantification of β-APP. (**E**) Yellow dashed line indicates the border between the white and gray matter of the spinal cord. Areas enclosed within white (**A**,**C**) are magnified on the right, respectively (**B**,**D**). The data are expressed as means ± SEM; *n* = 5 per group. Statistical significance was verified using ANOVA, with Bonferroni’s multiple comparisons test at a 0.05 confidence level (*** *p* < 0.001). Scale bars: (**A**,**C**) = 200 µm, and inset (**B**,**D**) = 50 µm.

Beta-APP, the main component of amyloid plaques in neurodegenerative diseases, promotes demyelination and axonal loss. β-APP accumulation was observed in the spinal cord white matter, both as larger amyloid plaques and small β-APP aggregates, in the area occupied by inflammatory cells (Figure 4A,B), as also revealed by CR staining. The β-APP expression level progressed from the onset phase to the peak phase (Figure 4E, Table 2) and was associated with demyelinating changes (Figure 2I, Table 1). However, as EAE progressed, the expression of β-APP remained stable between the peak phase and the chronic phase (Figure 4E). Control mice showed normal histology, with no β-APP expression (Figure 4C,D). 

**Figure 5:**
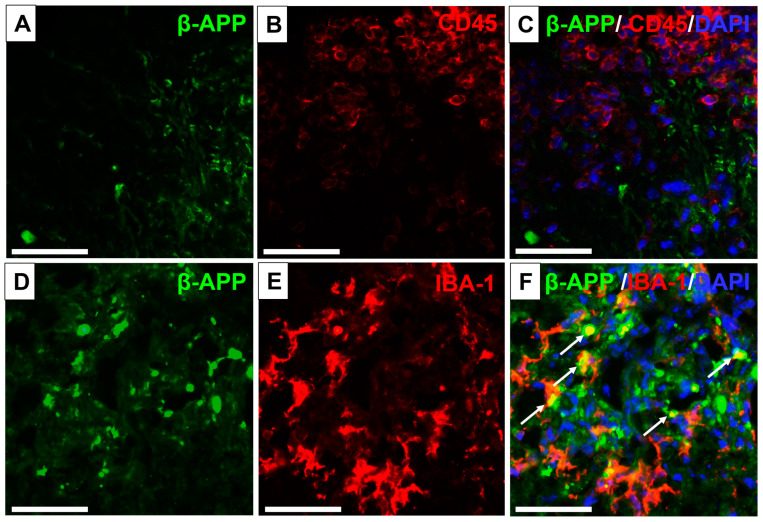
Area of inflammatory infiltration in the white matter of the spinal cord in the EAE peak phase. Immunostaining of β-APP (**A**,**D**), CD45 (**B**), and IBA-1 (**E**) and the overlap of β-APP/CD45 (**C**) and β-APP/IBA-1 (**F**), accompanied by DAPI nuclear staining (**C**,**E**). Colocalization for β-APP/IBA-1 ((**F**), white arrows). Scale bars: (**A**–**F**) = 50 µm.

Since β-APP was accumulated between inflammatory cells, CD45 and Iba-1 immunofluorescence labeling of leukocytes and macrophages/microglia, respectively, were also performed (Figure 5A–F). There was no colocalization of β-APP with CD45 (Figure 5A–C), but colocalization of β-APP with Iba-1 was visible in some macrophages/microglia (Figure 5D–F). Our previous research showed that the highest immunoreactivity of CD45 and Iba-1 was observed at the peak of neurological symptoms and was associated with the progression of EAE [17].

### 3.4. Tau/Phospho-Tau (p-Tau) Expression in EAE and Control Mice

**Figure 6:**
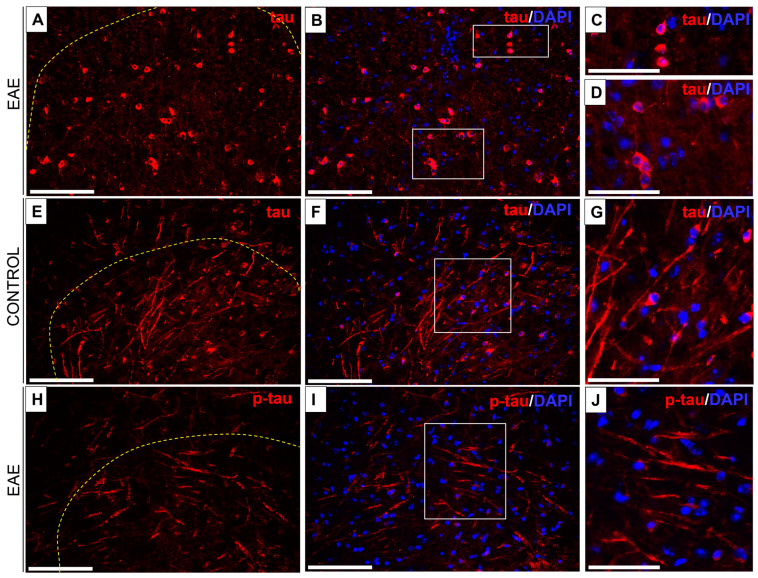
Tau (**A**–**D**) and p-tau (**H**–**J**) immunofluorescence in the chronic phase of EAE and tau expression in the control group (**E**–**G**). Tau is expressed in EAE mice, mainly in the perikaryons of nerve cells and in a few nerve fibers (**A**–**D**), and in control mice, it is expressed in both nerve fibers and nerve cell perikaryons (**E**–**G**). Immunolabeling of p-tau was observed predominantly in nerve fibers of EAE mice (**H**–**J**). A yellow dashed line indicates the border between the white and gray matter of the spinal cord. Areas enclosed within white (**B**,**F**,**I**) are magnified on the right, respectively (**C**,**D**,**G**,**J**). Nuclei were visualized by DAPI staining. Scale bars: (**A**,**B**,**E**,**F**,**H**,**I**) = 100 µm; insets (**C**,**D**,**G**,**J**) = 50 µm.

Tau/p-tau expression was demonstrated in both white and gray matter of the spinal cord of EAE mice (Figure 6A–J). In control mice, tau expression was observed in nerve fibers and nerve cell perikaryons, with a predominance of expression in nerve fibers (Figure 6E–G). A similar localization of immune expression occurred during the onset and peak phases in EAE mice, whereas in the chronic phase, tau was labeled mainly in the perikaryons of nerve cells (Figure 6A–D). However, p-tau immunostaining was seen only in nerve fibers (Figure 6H–J). No p-tau expression was observed in control mice.

**Figure 7:**
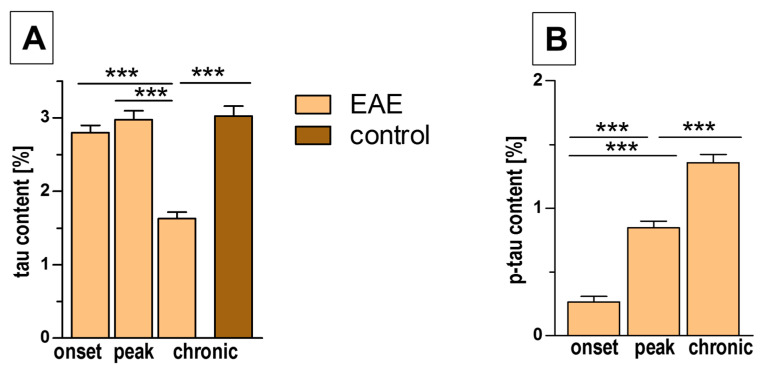
Tau (**A**) and p-tau (**B**) contents in the spinal cord at different phases of EAE. Tau expression was quantified as the percentage of the immunostained area in EAE mice in comparison to control mice. The data are expressed as means ± SEM; *n* = 5 per group. Statistical significance was verified using ANOVA with Bonferroni’s multiple comparisons test at a 0.05 confidence level (*** *p* < 0.001).

Immunofluorescence showed a significant decrease in tau expression in the chronic phase of EAE, whereas in the other phases, tau content was similar to that in control mice (Figure 7A, Table 2). The expression of p-tau increased with the progression of EAE, reaching a maximum in the chronic phase, which corresponded well with a similar increase in axonal loss (Figure 7B, Table 2).

### 3.5. Correlation of β-APP/Tau/p-Tau with Inflammation and Neurodegeneration Parameters

**Figure 8:**
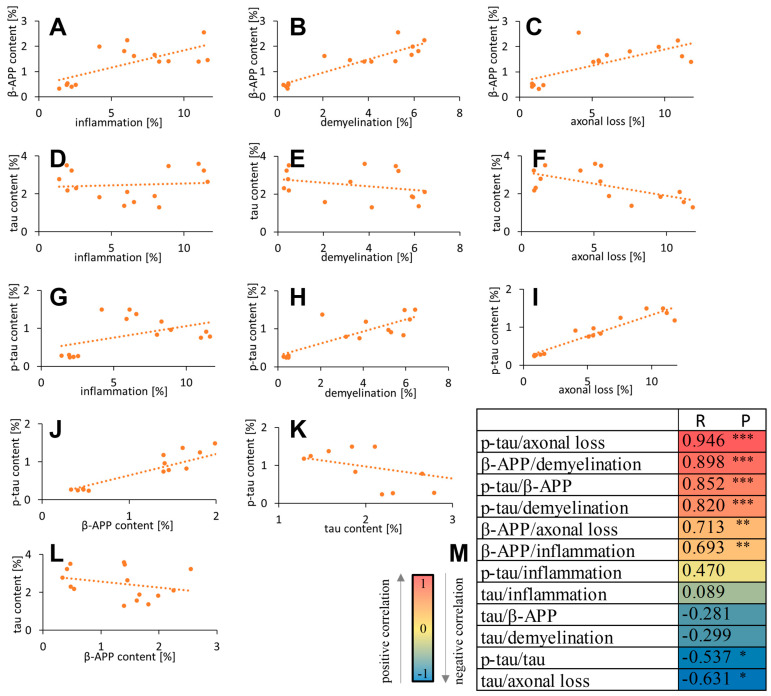
Correlations of β-amyloid (**A**–**C**), tau (**D**–**F**), and p-tau contents (**G**–**I**) with inflammation, demyelination, and axonal loss for EAE mice, as well as tau/p-tau content (**L**) with β-amyloid (**J**,**K**). The heatmap displays the Pearson correlation coefficients between APP, tau, p-tau, and histological parameters. * *p* < 0.05, ** *p* < 0.01, and *** *p* < 0.001 (**M**).

We calculated correlations between APP/tau/p-tau quantification and histological data (Figure 8A–M). Positive correlations were found between p-tau and axonal loss (R = 0.946, *p* < 0.0001), β-APP and demyelination (R = 0.898, *p* < 0.0001), p-tau and β-APP (R = 0.852, *p* < 0.0001), p-tau and demyelination (R = 0.820, *p* = 0.0002), β-APP and axonal loss (R = 0.713, *p* = 0.003), and β-APP and inflammation (R = 0.693, *p* = 0.004). P-tau and tau (R = −0.537, *p* = 0.039) and tau and axonal loss (R = −0.631, *p* = 0.012) were negatively correlated. There were no correlations between p-tau and inflammation (R = 0.470, *p* = 0.077), tau and inflammation (R = 0.089, *p* = 0.752), tau and β-APP (R = −0.281, *p* = 0.310), and tau and demyelination (R = −0.299, *p* = 0.279) (Figure 8A–M).

## 4. Discussion

The exact pathogeneses of Alzheimer’s disease and other neurodegenerative diseases are not fully understood. However, β-APP plaque deposition and tau neurofibrillary tangle aggregation are considered to be factors in the neuropathology of the disease [18]. The deposition of amyloid peptides in the CNS can cause a number of clinical problems in neurological and neurosurgical practice. Pathological expression of tau leads to a disturbance of the axoplasmic transport and contributes to neuronal degeneration.

In the present study, we used an EAE model, in which the progression of clinical severity was examined to characterize three sequential phases of the disease: onset, peak, and chronic. As also shown in previous studies, inflammation and neurodegeneration, as well as demyelination and axonal loss, are closely related to the progression of multifocal changes in the course of EAE [15,17,19,20,21]. These correlations were also demonstrated in the present study, which showed time-dependent changes in all main histopathological parameters of the disease. The levels and abundances of inflammatory infiltrates, demyelination, and axonal loss worsened with the increase in clinical severity scores, manifested as impaired motor function in mice.

Tau protein expression was observed during all phases of EAE progression, but its distribution pattern changed over time. In the onset and peak phases, tau was localized mainly in nerve fibers, whereas in the chronic phase, tau was mainly observed in the cell bodies of neurons. This redistribution of tau expression is consistent with results reported in other neurodegenerative diseases, including Alzheimer’s disease, in which tau proteins translocate from axons to the soma of nerve cells, impairing axonal transport and contributing to neuronal dysfunction [22]. In the EAE model, mice lacking tau proteins were found to be more susceptible to neuroaxonal damage, whereas mice expressing it had a more severe disease course and significantly greater axonal damage [23].

The observed increase in the p-tau level in the chronic phase of EAE seems to be the key finding of the present study. Elevated p-tau levels have been associated with decreased microtubule stability and impaired neuroaxonal transport, ultimately leading to neurodegeneration. The positive correlation between p-tau and axonal damage is consistent with previous studies that highlighted p-tau as a marker of neuronal damage in MS lesions. The accumulation of abnormal p-tau protein was associated with the loss of neurons and axons during the progression towards the chronic phase of EAE and in secondary progressive MS [24]. Enhanced p-tau expression is often observed in the later phases of MS, and it might result from the prion-like self-replication of p-tau molecules, causing the spread of the neurodegenerative processes, independent of the initial inflammatory triggers [25].

Interestingly, while the tau level significantly decreased in the chronic phase of EAE progression, p-tau expression continued to increase, indicated by a significant negative correlation. This change in trend during EAE progression suggests that p-tau accumulation may outweigh tau in terms of neurotoxicity, especially as far as long-term neurodegeneration is concerned. The phosphorylated form of tau is more likely to form neurofibrillary tangles, which can contribute to irreversible neuronal damage, as shown in other neurodegenerative diseases [9,24,26]. The decrease in tau content without a similar change in β-APP suggests a selective proteasomal degradation of tau protein.

Our study also highlighted the pathological role of β-APP in EAE progression. β-APP expression increased significantly from the onset to the peak phase, remaining stable during the chronic phase. β-APP is regarded as a marker of axonal damage but may also help to alleviate the effects of inflammation in neurodegenerative diseases such as MS and AD [14,15,17]. In this study, β-APP aggregates were found in inflammatory areas, being particularly associated with microglia and macrophages, which suggests a potential role of β-APP in perpetuating inflammation. Moreover, microglia can be responsible for the modification of extracellular neurofibrillary tangles [27]. The present results show that APP is colocalized with macrophages/microglial cells identified by Iba-1; colocalization with leukocytes identified by CD45 was never observed. Indeed, microglia have been shown to colocalize with β-APP, contributing to chronic inflammation and secondary neurodegeneration [27]. Interestingly, while β-APP strongly correlated with both demyelination and inflammation, it did not colocalize with CD45+ leukocytes, indicating that its role may be more closely associated with the activation of resident microglia/macrophages, rather than with infiltrating immune cells. This supports previous findings in MS, where β-APP accumulated in damaged axons and was associated with microglia activation but not directly with lymphocytic infiltration and could polarize microglia/macrophages toward an anti-inflammatory phenotype [14,27,28,29]. Furthermore, in AD, microglia have been shown to convert aggregated amyloid-beta into neurotoxic forms using excretion microvesicles [30].

Moreover, this study demonstrates a strong correlation between β-APP, axonal loss, and demyelination. This finding aligns with results concerning MS/AD diseases, where β-APP accumulation in axons has been linked to white matter damage and myelin loss [31,32]. The persistence of β-APP plaques and small aggregates in the chronic phase of EAE suggests that amyloidogenic processes continue beyond the acute inflammatory stage, contributing to ongoing axonal degeneration and neurological decline. We found significant correlations between β-APP and several neurodegenerative markers, including demyelination and axonal damage, highlighting its potential role as a mediator of neurodegeneration in EAE. Similarly, p-tau was positively correlated with axonal loss, demyelination, β-APP expression, and inflammation, indicating a pathological interplay between tau hyperphosphorylation and amyloidogenesis in promoting neurodegeneration.

Previous studies have postulated that β-APP accumulation precedes tau pathology, initiating a cascade of events that culminates in neuronal death [5,7]. Our data support this hypothesis in the context of EAE, as β-APP accumulation was observed early in disease progression, followed by tau and p-tau aggregation in later stages. Importantly, our findings suggest that once tau pathology is established, amyloid-targeted therapies may have limited effectiveness because tau tangles can independently cause neuronal damage.

Studies of MS lesions have demonstrated similar patterns of β-APP accumulation in early axonal injury and tau pathology in chronic lesions, highlighting the relevance of our findings to MS pathogenesis. Moreover, tau and p-tau may serve as biomarkers of the transition from relapsing-remitting MS to progressive MS, in which neurodegenerative processes become more pronounced. Tau-targeted therapies already under investigation for AD could potentially be used to treat progressive MS, which is dominated by inflammation-independent neurodegeneration.

This study provides an analysis of tau and β-APP in the context of neuroinflammation and neurodegeneration in the EAE model of MS. The correlation between these proteins and the classical markers of EAE/MS pathology highlights their potential roles in the progression of neurodegenerative mechanisms in MS and suggests that targeting these proteins may offer new therapeutic opportunities, particularly in progressive phases of the disease. Future studies focused on modulating tau phosphorylation and β-APP accumulation may provide valuable clues about the possibility of halting or even reversing neurodegeneration in MS.

## Figures and Tables

**Table 1:** Clinical scores and pathological parameters of EAE assessed in the spinal cord of EAE mice during the progression of the disease. Data expressed as mean ± SEM were determined for 5 mice per phase.

EAE Mice	Onset Phase	Peak Phase	Chronic Phase
**day post-immunization**	14	19	30
**mean EAE score**	1.02 ± 0.05	2.97 ± 0.15	1.83 ± 0.09
**cumulative score**	-	-	36.52 ± 1.83
**number of inflammatory foci per cross-section**	1.88 ± 0.17	8.52 ± 0.22	6.84 ± 0.23
**inflammation area [%]**	2.02 ± 0.11	10.19 ± 0.48	6.20 ± 0.33
**demyelination area [%]**	0.41 ± 0.03	4.67 ± 0.28	4.95 ± 0.30
**axonal loss area [%]**	1.12 ± 0.11	5.21 ± 0.21	10.21 ± 0.38

**Table 2:** Expressions of β-amyloid and tau/p-tau in the spinal cord of EAE and control mice in the successive phases of the disease. Data expressed as mean ± SEM were determined for 5 mice per group/phase.

	EAE			Control
	Onset Phase	Peak Phase	Chronic Phase	
**β-amyloid content [%]**	0.44 ± 0.03	1.69 ± 0.13	1.81 ± 0.11	-
**tau content [%]**	2.80 ± 0.10	2.98 ± 0.12	1.63 ± 0.09	3.02 ± 0.14
**p-tau content [%]**	0.26 ± 0.04	0.85 ± 0.05	1.36 ± 0.07	-

## Data Availability

The original contributions presented in this study are included in the article. Further inquiries can be directed to the corresponding author.
